# Osteoblasts contribute to a protective niche that supports melanoma cell proliferation and survival

**DOI:** 10.1111/pcmr.12812

**Published:** 2019-08-08

**Authors:** Jennifer Ferguson, Daniel J. Wilcock, Sophie McEntegart, Andrew P. Badrock, Mitch Levesque, Reinhard Dummer, Claudia Wellbrock, Michael P. Smith

**Affiliations:** ^1^ Manchester Cancer Research Centre, Faculty of Biology, Medicine and Health The University of Manchester Manchester UK; ^2^ Department of Dermatology, Universitäts Spital Zürich University of Zürich Zurich Switzerland

**Keywords:** BRAF, melanoma, MITF, Osteoblasts, RANKL, resistance

## Abstract

Melanoma is the deadliest form of skin cancer; a primary driver of this high level of morbidity is the propensity of melanoma cells to metastasize. When malignant tumours develop distant metastatic lesions the new local tissue niche is known to impact on the biology of the cancer cells. However, little is known about how different metastatic tissue sites impact on frontline targeted therapies. Intriguingly, melanoma bone lesions have significantly lower response to BRAF or MEK inhibitor therapies. Here, we have investigated how the cellular niche of the bone can support melanoma cells by stimulating growth and survival via paracrine signalling between osteoblasts and cancer cells. Melanoma cells can enhance the differentiation of osteoblasts leading to increased production of secreted ligands, including RANKL. Differentiated osteoblasts in turn can support melanoma cell proliferation and survival via the secretion of RANKL that elevates the levels of the transcription factor MITF, even in the presence of BRAF inhibitor. By blocking RANKL signalling, either via neutralizing antibodies, genetic alterations or the RANKL receptor inhibitor SPD304, the survival advantage provided by osteoblasts could be overcome.

**Significance**

Understanding how specific tissue niches leads to resistance to melanoma therapies is essential to generating robust and sustainable patient responses. By looking at the signalling between melanoma cells and osteoblasts we have characterized a mechanism with the potential to provide resistance to BRAF inhibitors that is specific to the bone niche. The output of this investigation has identified a RANKL‐MITF signalling axis that can be targeted to antagonize osteoblast contributions towards resistance.

## 1 INTRODUCTION

Melanoma is the deadliest form of skin cancer, and arises from the transformation of pigment cells in the skin called melanocytes. Poor prognostic outcomes in melanoma are associated with resistance to conventional therapy and the highly metastatic nature of melanoma (Sandru, Voinea, Panaitescu, & Blidaru, 2014), whereby multiple tissue‐specific niches are capable of being populated by this transformed cell lineage. These niches provide unique signalling environments that are co‐opted by the melanoma to promote growth and survival. Therefore, when studying the interface between therapies and basic melanoma biology, it is important to understand the cellular and tissue context of cancer cells. This is exemplified by the Paget‐Seed‐Soil theorem that states the tissue site (soil) that a cancer (seed) preferentially metastasizes to is pre‐defined by mutualistic factors (Fidler, 2003).

One secondary site that appears to provide strong support for cancer cells is the bone (Bubendorf et al., 2000; Rahim et al., 2014). For instance, up to 90% of prostate cancers that metastasize will spread to the bone; an event that predicates poor survival independent of therapeutic intervention (Bubendorf et al., 2000). The stromal make‐up of the bone is rich in endothelial and immune cells, though the deposition of extracellular matrix (ECM) is chiefly controlled by two cell lineages, osteoblasts and osteoclasts (Park, Keller, & Shiozawa, 2018). Osteoclasts breakdown and remodel the mineralized bone matrix, while osteoblasts function to produce new ECM in addition to controlling the number and activation of osteoclasts. The factors that drive the homing and aggressive growth of bone metastases are well appreciated, and include abundant supply of blood and oxygen, and a wealth of secreted factors that emanate from bone stroma, including IGFs, BMPs, FGFs, PDGF and RANKL (Park et al., 2018; Rahim et al., 2014; Sottnik & Keller, 2013; Zheng, Li, & Kang, 2016). RANKL is of particular interest because it stimulates osteoclastogenesis, which in turn drives the loss of bone matrix; an event often observed in patients with bone metastases (Hegemann, Bedke, Stenzl, & Todenhöfer, 2017). Indeed, the RANKL inhibitor Denosumab both decreases the rate of bone degeneration by inhibiting osteoclastogenesis, and diminishes the spread of prostate cancer (Hegemann et al., 2017). RANKL has also been shown to play a role in the ability of melanoma cells to home to, and grow within the bone niche (Jones et al., 2006).

Melanoma cell growth is typically driven by the ERK/MAPK pathway, which is hyper‐activated in at least 80% of melanomas that occur through mutations in *NRAS* (~20%), *BRAF* (~50%) and *NF1* (~14%) (Akbani et al., 2017). Therefore, monotherapies using a BRAF inhibitor (BRAFi) or combination therapies of BRAF and MEK inhibitors (MAPKi) are now considered a mainstay of melanoma treatment (Long et al., 2015). However, maintaining initial responses are problematic due to the development of resistance driven by a plethora of mechanisms (Arozarena & Wellbrock, 2017; Smith & Wellbrock, 2016). We have shown previously that the master regulator of survival, growth and differentiation in pigment cells, MITF, contributes to resistance by increasing tolerance to MAPKi during initial treatment (Smith et al., 2016, 2017). This occurs in concert with alterations in surrounding tumour stroma that further decreases response to therapy (Smith et al., 2014; Wang et al., 2015; Young et al., 2017), and involves fibroblasts, macrophages and even the ECM (Hirata et al., 2015; Qin et al., 2016; Straussman et al., 2012). The variable composition of the stroma between potential metastatic sites suggests the possibility of differential responses to therapy. Indeed, melanomas located either in bone lesions or the Central Nervous System (CNS) have worse response rates to MAPKi therapy (16%) compared to all other sites (>70%) (Seifert et al., 2016). Additionally, mutations that drive resistance within a relapsed patient differ between metastatic sites (Kemper et al., 2015).

While secreted factors found in the cerebrospinal fluid are known to contribute to the CNS‐induced therapy resistance of melanomas (Seifert et al., 2016), the contribution of the bone‐specific stromal niche to resistance to targeted therapies is unknown. Thus, we examined signalling between melanoma and osteoblasts, and the role of this interplay in MAPKi resistance.

## 2 MATERIALS AND METHODS

### 2.1 Cell Culture and drug treatments

Melanoma cell lines were grown in DMEM/10% Fetal Calf Serum (FCS) (PAA, Yeovil, UK). Human melanocytes were from Cascade Biologics and grown according to manufacturer´s guidelines. PD184352 was from Axon Medchem, (Groningen, The Netherlands); AZD6244 and vemurafenib were from Selleck Chemicals (Newmarket, UK). SPD304 was acquired from Sigma (St Louis, MO, USA). Recombinant human PTH and RANKL were acquired from PeproTech (London, UK). The MITF status of cell lines used in this study is: MITF negative – SKMEL105, MITF low – A375, WM266‐4 MITF high – 501MEL, WM164, WM98 (Smith et al., 2016). Conditioned medium (CM) was generated by incubating cells for 24 hr with fresh culture medium containing FCS was then filtering (0.45 µm) to remove cells and debris.

### 2.2 Osteoblast differentiation and co‐culture

Osteoblast precursor cells hFOB 1.19 were acquired from ATCC (CRL‐11372). hFOB 1.19 cells were cultured at 34°C in HAMs F12 medium and DMEM/10% FCS (PAA, Yeovil, UK) at a ratio of 1:1 in a humidified 5% CO2 incubator. Differentiation was performed by transferring cells to 39°C in a humidified 5% CO2 incubator and supplementing media with either filtered CM from melanoma cells or spiked with recombinant PTH. For co‐culture assays, hFOB 1.19 cells were differentiated in transwell inserts (BD Biosciences) and washed 3x with DMEM before they were incubated with melanoma cells. For direct co‐culture experiments individual cultures of 0.2 × 105 osteoblasts and 0.5 × 105 A375 cells, respectively were stained and quantified and compared to a co‐culture of 0.2 × 105 osteoblasts and 0.5 × 105 A375 cells.

### 2.3 RNA interference

Specific mRNA depletion was performed using RANK siRNA: GAACCAGGAAAGUACAUGU, MITF siRNA: MITF #001 GAACGAAGAAGAAGAUUUAUUU, #003 AAAGCAGUACCUUUCUACCAC. Control si‐control AAUAUAAUCACUAUCAGGUGC. All siRNAs were transfected using Interferin (Polyplus, Illkirch, France) following the manufacturer's instructions.

### 2.4 RNA isolation and RT‐qPCR analysis

RNA from cell lines was isolated with TRIZOL® and selected genes were amplified by quantitative real time PCR (RT‐qPCR) using SYBR green (Qiagen, Valencia, CA, USA). Relative expression was calculated using the delta‐delta CT methodology and beta‐actin was used as reference housekeeping gene (Livak & Schmittgen, 2001).

### 2.5 Primers sequences for SYBR green RT‐qPCR were

MITF, 5′‐CCGTCTCTCACTGGATTGGT‐3′, 5′‐TACTTGGTGGGGTTTTCGAG‐3′; TRPM1, 5′‐CACCCAGAGCTACCCAACAGA‐3′, 5′‐CGGATATACATGGCTTTATTGG‐3′; PMEL, 5′‐CTGGATGGTACAGCCACCTT‐3′, 5′‐GGCACTTTCAATACCCTGGA‐3′; TYR, 5′‐CTGGAAGGATTTGCTAGTCCAC‐3′, 5′‐CCTGTACCTGGGACATTGTTC‐3′; CCND1, 5′‐GAACTACCTGGACCGCTTCCT‐3′, 5′‐TTCGATCTGCTCCTGGCAGG‐3′; BCL2, 5′‐CGCCCTGTGGATGACTGAGT‐3′, 5′‐CCCAGCCTCCGTTATCCTG‐3′; BCL2A1, 5′‐CGTAGACACTGCCAGAACACTA‐3′, 5′‐GGGCAATTTGCTGTCGTAGA‐3′; B‐ACTIN: 5′‐GCAAGCAGGAGTATGACGAG‐3′, 5′‐CAAATAAAGCCATGCCAATC‐3′; PTHRP, 5’‐TTTACGGCGACGATTCTTCC‐3’, 5’‐ TTCTTCCCAGGTGTCTTGAG‐3’; SPP1, 5’‐ACTGATTTTCCCACGGACCT‐3’, 5’‐GGATGTCAGGTCTGCGAAAC‐3’; RANKL, 5’‐GTGCAAAAGGAATTACAACATATCGT‐3’, 5’‐ AACCATGAGCCATCCACCAT‐3’; RANK, 5’‐ TGGAGAAGCACAGGACAGTT‐3’, 5’‐ AGGGCAGGAATGACGGTAAA‐3’. MLANA, 5′‐TCTGGGCTGAGCATTGGG‐3′, 5′‐AGACAGTCACTTCCATGGTGTGTG‐3′; CDK2, 5′‐ATGGAGAACTTCCAAAAGGTGGA‐3′, 5′‐CAGGCGGATTTTCTTAAGCG‐3′;

### 2.6 EdU incorporation

Cells were labelled with 20 µM 5‐ethynyl‐2'‐deoxyuridine (EdU) for 4h and processed following the manufacturer's protocol (Click‐iT^®^ EdU Alexa Fluor^®^ 488 Imaging Kit, Thermo Fisher). Stained cells were analysed using a BDpathway 855 Bioimager.

### 2.7 IncuCyte caspase analysis

To assess apoptosis induction, 5,000 cells per well were seeded in a black 96 well tissue culture plate (BD Falcon, SLS). IncuCyte Kinetic Caspase‐3/7 Apoptosis Assay Reagent (Essen BioScience) was added at a dilution of 1/10,000. If caspase 3/7 had been activated apoptotic cells could be detected by a fluorescence signal. Cells were kept at 37°C in a humidified 5% CO2 incubator and imaged using an IncuCyte ZOOM (Essen BioScience) and a 20 × lens.

### 2.8 Cell lysis and immunoblotting

Cells were lysed in SDS sample buffer and analysed by standard Western‐blotting protocols. The primary antibodies used were: phospho‐ERK (MAPK‐YT) and BCL2 (10C4) from Sigma (St Louis, MO, USA); ERK2 (C‐14) and BETA‐TUBULIN were from Santa Cruz Biotechnology (Santa Cruz, CA, USA); MITF (C5) was from Neomarkers/Lab Vision (Runcorn, UK); phospho‐P65 (93H1) and phospho‐P38 (D3F9) were from Cell Signalling (Danvers, MA, USA); RANK (9A725) was from Novus Biologicals (Abingdon, UK); RANKL (MAB626) neutralizing antibody was used at 40ng/ml and was from R&D systems (Minneapolis, MN, USA).

### 2.9 Statistical analysis

If not indicated otherwise, data represent the results for assays performed in triplicate, with error bars to represent *SEM*. Statistics used were: predominately Student *t* test and one‐way ANOVA with Tukeys's post hoc test performed using GraphPad Prism version 7.00 for Mac OS, GraphPad Software (San Diego California USA).

## 3 RESULTS

### 3.1 Differentiated osteoblasts enhance melanoma growth and differentiation

**Figure 1:**
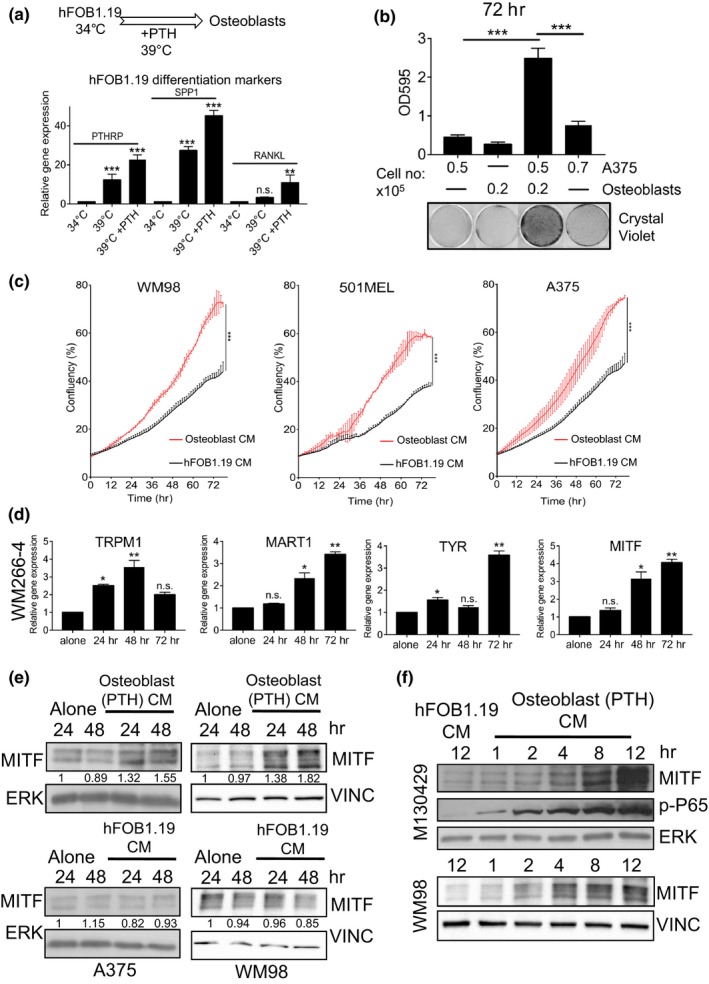
Osteoblasts support melanoma growth and enhance differentiation. (a) Overview of culture conditions of hFOB1.19 cells, and RT‐qPCR analysis of indicated gene expression. (Mean ± *SEM*, *n* = 5) (b). Image and quantification of crystal violet stained A375 cells and PTH‐differentiated osteoblast when either in mono‐ or co‐culture for 72 hr. (Mean ± *SEM*, *n* = 3) (c). Growth curves of indicated cell lines with or without conditioned medium from PTH‐differentiated osteoblasts determined by time‐lapse microscopy using an Incucyte system (Mean ± *SD*, *n* = 3) (d). Relative gene expression of indicated genes in WM266‐4 cells with or without conditioned medium from PTH‐differentiated osteoblasts at stated time intervals. (Mean ± *SEM*, *n* = 3) (e). Protein expression of MITF and ERK in indicated cell lines with or without conditioned medium from hFOB1.19 or PTH‐differentiated osteoblasts at stated time intervals. Relative MITF expression was quantified using Image J and normalized to ERK. (f) Protein expression of MITF, phospho‐P65 and ERK in short‐term cultured M130429 cells and WM98 with or without conditioned medium from hFOB1.19 or PTH‐differentiated osteoblasts at stated time intervals

To investigate whether a niche of bone stromal cells interacts with melanoma cells we utilized a model of differentiating osteoblast precursor cells hFOB 1.19 as previously described (Harris, Enger, Riggs, & Spelsberg, 2009). Transfer of hFOB 1.19 cells to 39°C and subsequent treatment with PTH over 7 days led to increased expression of differentiated osteoblast markers such as PTHrP, SPP1 and RANKL (Figure 1a). Direct co‐culture of melanoma cells with differentiated osteoblasts (Figure S1a) caused a significant increase in melanoma cell numbers over 72 hr (Figure 1b), indicating potential cross‐talk between the two cell types. To determine whether the pro‐growth effect produced by osteoblasts was based on a secreted factor, or a result of direct cell‐cell contact, growth assays were performed using osteoblast conditioned medium (CM). Several melanoma cell lines showed an increase in cell numbers in response to treatment with osteoblast‐CM, an effect not observed with undifferentiated hFOB 1.19 CM (Figure 1c). The pro‐growth effect of osteoblast‐CM was replicated in M130429 cells, a short‐term culture, isolated from a bone metastatic tumour (Figure S1b).

The pro‐growth effect in response to osteoblast‐secreted factors was linked to enhanced expression of genes related to cell‐cycle progression and pigment cell differentiation (Figure 1d, Figure S1c). Many of these genes are MITF targets and accordingly, MITF expression was increased (Figure 1d). When the protein levels of MITF were analysed, only CM from differentiated osteoblasts was able to enhance MITF expression; CM from precursor hFOB 1.19 cells failed to elicit any change in MITF protein expression (Figure 1e). Together, these data suggest that MITF may be orchestrating the effects on melanoma cell differentiation and proliferation induced by osteoblasts.

Osteoblast cells are known to secrete many pro‐inflammatory cytokines that activate the NFκB family, and activation of RelA/p65 has previously been linked to MITF expression downstream of TNFα (Chu et al., 2014; Smith et al., 2014). Indeed, we observed that osteoblast‐CM induced phosphorylation of p65 in melanoma cells within hours and this correlated with increased MITF protein levels (Figure 1f).

### 3.2 Melanoma cells can differentiate osteoblasts and enhance RANKL expression

**Figure 2:**
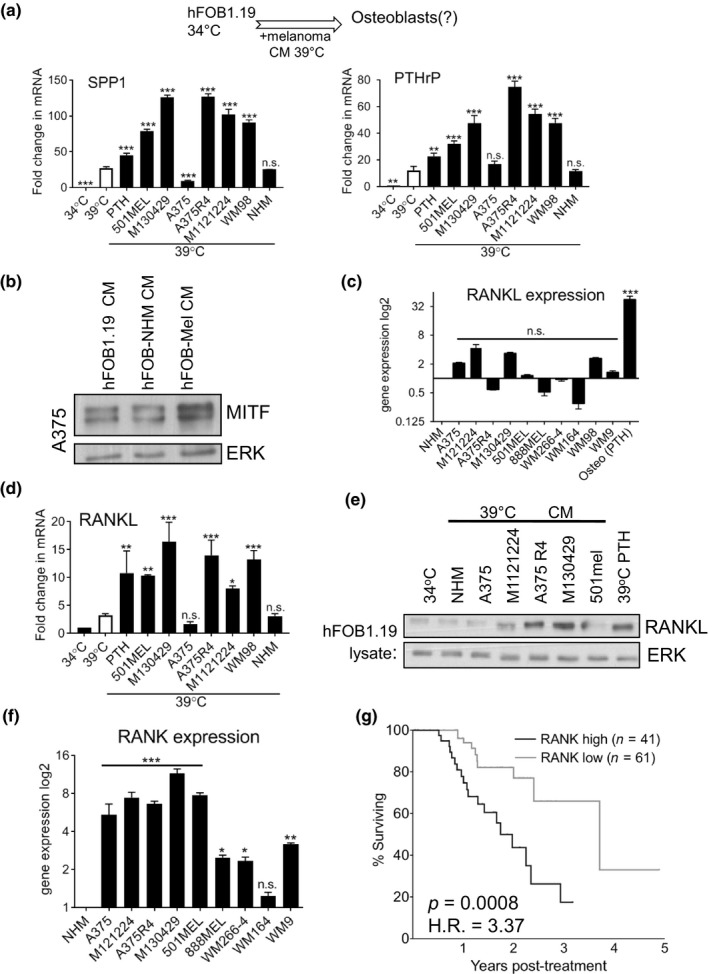
Melanoma cells induce RANKL expression in osteoblasts, and RANK expression correlates with poor survival (a). RT‐qPCR analysis of SPP1 and PTHrP expression in hFOB1.19 cells incubated at 39°C with CM from indicated melanoma cells. Relative expression was compared to 39°C hFOB1.19. (white bar) (Mean ± *SEM*, *n* = 3). An overview of the culture conditions is shown above the graphs (b). Protein expression of MITF and ERK in A375 cells after 72 hr of treatment with or without CM from hFOB1.19 or osteoblasts differentiated using NHM or M130429 CM (c). RT‐qPCR analysis of RANKL gene expression in indicated cell lines relative to NHM (Mean ± *SEM*, *n* = 3) (d). RT‐qPCR analysis of RANKL expression in hFOB 1.19 cells incubated with indicated melanoma CM (Mean ± *SEM*, *n* = 3) (e). Protein expression of RANKL and ERK in hFOB1.19 cells incubated with indicated melanoma CM. (f) RT‐qPCR analysis of RANK gene expression in indicated cell lines relative to NHM (Mean ± *SEM*, *n* = 3). (g) Patient survival analysis from subset of TCGA cohort of melanoma patients RNAseq expression data separated by RANK levels. Hazard Ratio calculated using Mantel‐Haenszel 3.37 with C.I 1.734 – 8.102, median survival 636 versus 1,354 days

We have recently shown that melanoma cells can promote the differentiation of macrophages (Young et al., 2017), as well as stimulate fibroblasts to alter the stiffness of their deposited ECM (Miskolczi et al., 2018). Similarly, we tested whether melanoma cells directly impact osteoblast differentiation by exposing the latter to melanoma cell CM. As observed for PTH‐differentiated osteoblast cells, incubation of hFOB 1.19 precursors with melanoma‐CM was sufficient to increase expression of the osteoblast markers PTHrP and SPP1 (Figure 2a). This response was not elicited by CM taken from normal human melanocytes (NHM), suggesting that secreted osteoblast differentiation factors are enriched in melanoma cells (Figure 2a). This ability to enhance osteoblast differentiation varies between melanoma cell‐lines indicating that this effect is not universal any may relate to unique secreted factors.

To next examine whether melanoma CM differentiated osteoblasts function in a similar way to PTH‐differentiated osteoblasts, we treated melanoma cells with the CM taken from osteoblasts that were differentiated with melanoma CM and we observed an increase in MITF expression (Figure 2b), as previously seen for CM taken from PTH‐differentiated osteoblasts (Figure 1d‐f).

So far we observed that CM from differentiated osteoblasts not only induces MITF expression, but also activates NFκB signalling in melanoma cells (see Figure 1f). A cell‐type‐restricted NFκB activator expressed at high levels in differentiated osteoblasts is RANKL (Leibbrandt & Penninger, 2008). Indeed, we found that RANKL expression was high in in vitro differentiated osteoblasts, but not significantly high in melanoma cells (Figure 2c). Moreover, we found that RANKL expression was up‐regulated at both RNA and protein level, in the melanoma CM‐differentiated osteoblasts (Figure 2d,e), suggesting that it could contribute to the induction of MITF expression in melanoma cells (see Figure 2b).

In order to respond to RANKL, melanoma cells need to express its receptor RANK. Indeed, RANK expression is significantly increased in transformed melanoma cell lines when compared to normal human melanocytes (Figure 2f, Figure S2a). Strikingly, higher expression of RANK in melanoma biopsies correlated with a significant decrease in overall survival, highlighting the potential importance of this signalling node (Figure 2g).

### 3.3 RANKL enhances proliferation and differentiation via MITF induction

**Figure 3:**
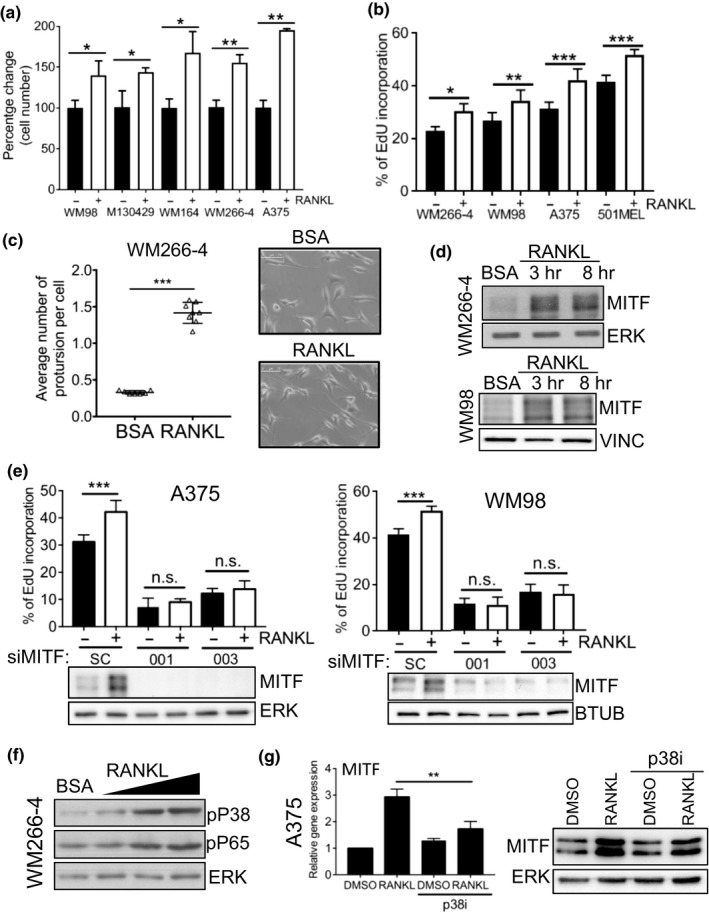
RANKL enhances proliferation via elevated MITF expression. (a) Quantification of crystal violet staining in indicated melanoma cell lines treated with/without 50ng/ml of RANKL for 72 hr. (Mean ± *SEM*, *n* = 3) (b). Determination of S‐phase population in indicated cell lines using EDU incorporation 24 hr after treatment with/without 50ng/ml of RANKL. Data are presented as percentage of EdU positive cells with DAPI as reference. (Mean ± *SEM*, *n* = 7) (c). Number of protrusions per cell in WM266‐4 cells treated with/without 50ng/ml of RANKL for 72 hr calculated by manual annotation of bright field images using Image J. (Mean ± *SEM*, *n* = 7) (d). Protein expression of MITF and ERK in indicated melanoma cell lines treated with/without 50ng/ml of RANKL for indicated time points (e). Protein expression of MITF, ERK and determination of S‐phase population using EDU incorporation (Mean ± *SEM*, *n* = 3) in indicated melanoma cell lines treated with/without 50ng/ml of RANKL for 24 hr. 24 hr before RANKL addition cells had been transfected with the indicated siRNAs (f). Protein expression of phospho‐P65, phospho‐P38 and ERK in WM266‐4 cells treated with 25ng/ml, 50ng/ml, 100ng/ml of RANKL for 24 hr (g). RT‐qPCR analysis of MITF mRNA expression and protein expression of MITF and ERK in A375 cells treated for 24 hr with 50ng/ml of RANKL with/without 10 μM p38 MAPK inhibitor SB203580 (Mean ± *SEM*, *n* = 3)

Our data suggested that although melanoma cells may not secrete significant levels of RANKL compared to osteoblasts, they should be competent to respond to RANKL in a tumour environment. To test this, we treated melanoma cells with exogenous RANKL. In a panel of melanoma cell lines exogenous RANKL induced a significant increase in cell number (Figure 3a) that correlated with increased EdU incorporation (Figure 3b). RANKL also stimulated the formation of cellular protrusions (Figure 3c) indicative of pigment cell differentiation (Nguyen & Fisher, 2019). As MITF is a known regulator of both differentiation and proliferation in melanoma cells, we examined MITF expression in RANKL‐treated melanoma cells. Addition of RANKL was sufficient to increase MITF expression after 3h (Figure 3d), and expression of markers of pigment cell differentiation (Figure S2b). It was striking to note that the effects of RANKL were broadly similar (Figure 3a,b) when comparing a number of melanoma cell lines, known to express different levels of MITF (Smith et al., 2016).

To further characterize the RANKL‐RANK‐MITF signalling axis, we first used RNAi to deplete MITF in melanoma cells prior to RANKL stimulation (Figure 3e). EdU incorporation assays demonstrated that MITF depletion blocked RANKL‐induced proliferation effects (Figure 3e, Figure S3a), indicating that RANKL acts through MITF. Furthermore, previous reports have shown that the p38 and NFκB pathways lie upstream of RANKL‐induced MITF expression in osteoclasts (Carey et al., 2016; Mansky, Sankar, Han, & Ostrowski, 2002; Vaira et al., 2008). Both p38 and p65 were found to be phosphorylated downstream of RANKL in melanoma cells (Figure 3f). To test whether the RANKL‐induced p38 activity was acting upstream of MITF, as reported for osteoclasts, we inhibited p38 and examined MITF expression. p38 inhibition partially reduced the RANKL‐induced increase in MITF protein levels, (Figure 3g right‐hand panel) consistent with a reduction in MITF mRNA abundance (Figure 3g left‐hand panel).

### 3.4 RANKL mediates survival through MITF

**Figure 4:**
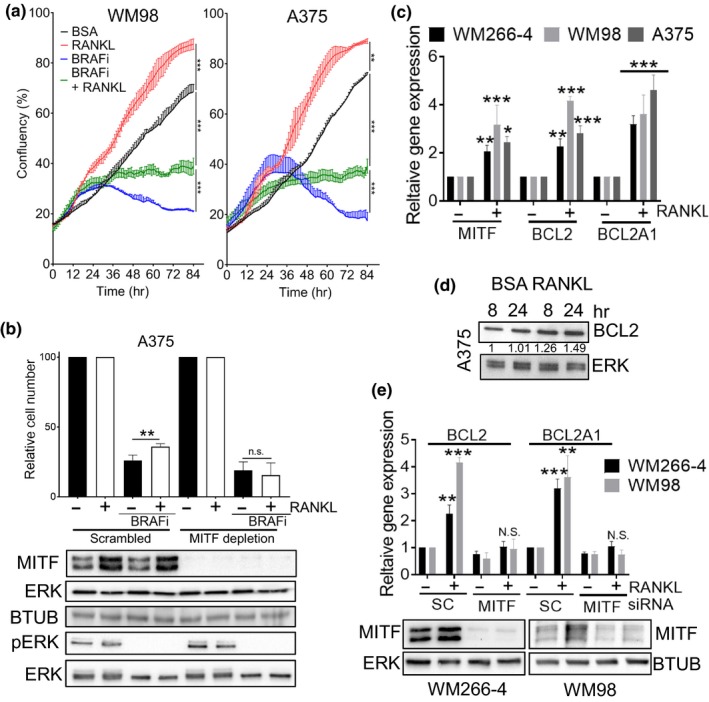
RANKL diminishes BRAF inhibitor activity via MITF‐induced survival signalling (a). Growth curves of indicated cell lines with or without BRAFi (vemurafenib 1 µM WM98, 0.5 µM A375) in the presence or absence of RANKL (50 ng). Confluency was measured by time‐lapse microscopy using an Incucyte system (Mean ± *SD*, *n* = 3) (b). Protein expression of phospho‐ERK, MITF, beta tubulin (BTUB) and ERK, and quantification of relative cell number as determined by crystal violet staining of A375 cells. Cells were treated for 72 hr with/without 50ng/ml RANKL, the final 48 hr cells were treated with either DMSO or 0.5 µM vemurafenib in the presence of either a Scrambled control or a MITF specific siRNA (20 nM). (Mean ± *SEM*, *n* = 3) (c). RT‐qPCR analysis of gene expression in indicated melanoma cell lines treated for 24 hr with/without 50ng/ml of RANKL. (Mean ± *SEM*, *n* = 4) (d). Protein expression of BCL2 and ERK in A375 cells with/without 50ng/ml of RANKL at indicated time points. Relative BCL2 expression was quantified using Image J and normalized to ERK (e). Protein expression of MITF, ERK and RT‐qPCR analysis of gene expression in indicated melanoma cell lines, treated for 24 hr with/without 50ng/ml of RANKL, following previous 24 hr transfection with indicated siRNA. (Mean ± *SEM*, *n* = 3)

It is well established that on treatment with MAPK inhibitors, increased MITF expression mediates melanoma cell survival (Haq, Yokoyama, et al., 2013; Johannessen et al., 2013; Rose et al., 2016; Smith et al., 2013, 2016, 2017; Song et al., 2017). Having identified RANKL as a secreted factor that increases MITF expression, combined with evidence that RANKL signalling is linked to survival of patients in other cancers (de Groot, Appelman‐Dijkstra, van der Burg, & Kroep, 2018), we next wanted to assess the role of RANKL in mediating resistance to BRAF inhibition. RANKL treatment was sufficient to increase cell numbers in BRAFi‐treated melanoma cells when compared to BRAFi treatment alone (Figure 4a, Figure S4a). Co‐treatment of RANKL and BRAFi had no effect on the loss of activated ERK compared to BRAFi alone, indicating that the increase in cell number was not mediated via re‐activation of the MAPK pathway (Figure S4a); a mechanism reported for other secreted factors such as HGF and EDN1 (Smith et al., 2017; Straussman et al., 2012).

To examine whether MITF mediated the RANKL‐dependent increase in melanoma cell number in the presence of BRAFi, cells were depleted of MITF expression using RNAi. Depletion of MITF abolished the increase in cell number mediated by RANKL (Figure 4b Figure S4b). The protective effect of RANKL in the presence of BRAFi is correlated with an increase in MITF pro‐survival target genes (Figure 4c,d), which is lost when MITF is depleted (Figure 4e). Co‐treatment of MITF negative melanoma cells with RANKL and BRAFi conferred no survival advantage (Figure S4c). Altogether, these data support an MITF‐dependent role for the RANKL‐mediated increase in melanoma survival upon BRAF inhibition.

### 3.5 Osteoblasts antagonize BRAF inhibition via RANKL secretion

Complex stromal and tumour cell interactions have previously been shown to be potent drivers of resistance during MAPK inhibitor therapies through the secretion of different growth factors such as TNFα, HGF and others (Hirata et al., 2015; Lito et al., 2012; Smith et al., 2014; Straussman et al., 2012). We had observed that CM from melanoma cells induced RANKL expression in differentiating osteoblast (Figure 2c‐d), and so we next asked whether secreted RANKL from osteoblasts is sufficient to provide melanoma cells with a survival advantage.

**Figure 5:**
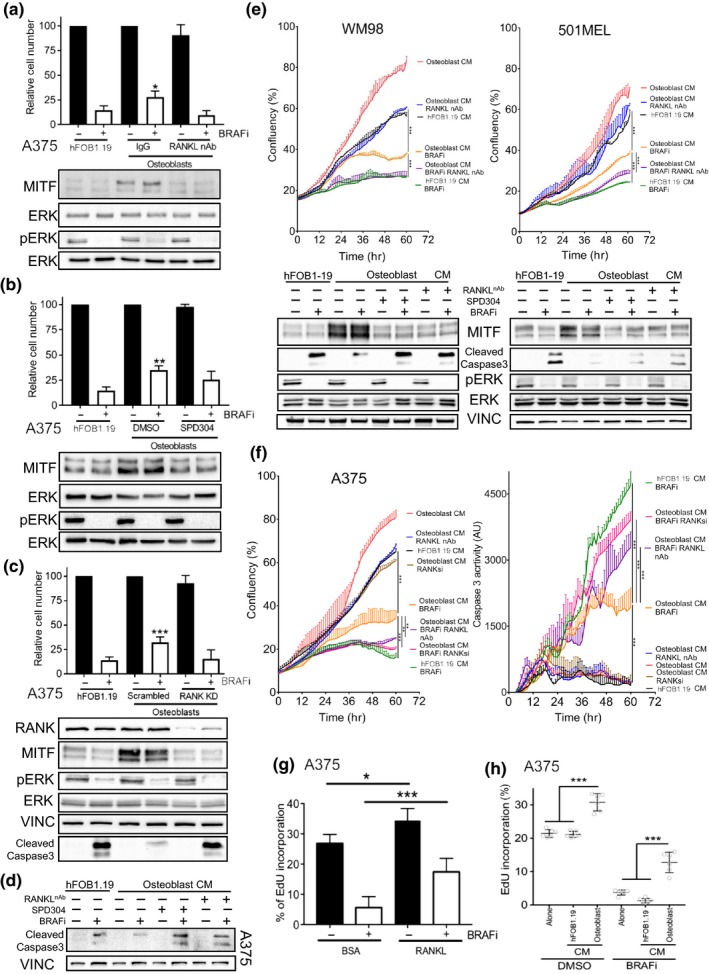
Osteoblasts provide a BRAF inhibitor protective niche via RANKL secretion (a). Relative cell number and protein expression of MITF, phospho‐ERK, and ERK in A375 cells following 72 hr treatment with/without 0.5 µM vemurafenib. Cells were co‐cultured with either hFOB1.19 or osteoblast in transwells with addition of 40ng/ml RANKL nAb or IgG. (Mean ± *SEM*, *n* = 7) (b). Relative cell number and protein expression of MITF, phospho‐ERK, and ERK in A375 cells co‐cultured with either hFOB1.19 or osteoblasts in transwells and treated for 72 hr with/without 0.5 µM vemurafenib in addition of 10 µM SPD304. (Mean ± *SEM*, *n* = 7) (c). Relative cell number and protein expression of MITF, phospho‐ERK, RANK, VINCULIN, cleaved‐CASPASE3 and ERK in A375 cells; following 72 hr treatment with/without 0.5 µM vemurafenib grown in the presence of co‐culture transwells with either hFOB 1.19 or osteoblasts. Melanoma cells had either been transfected with a Scrambled control or a RANK specific siRNA (Mean ± *SEM*, *n* = 3) (d). Protein expression of VINCULIN and Cleaved‐CASPASE3 in A375 cells; following 72 hr treatment with/without 0.5 µM vemurafenib grown in incubated in indicated CM from either hFOB 1.19 or osteoblasts, in the background of 40ng/ml RANKL nAb or 10 µM SPD304 (e). Growth curves of indicated cell lines with or without BRAFi (vemurafenib 1 µM) incubated in indicated CM from either hFOB 1.19 or osteoblasts, in the background of 40ng/ml RANKL nAb. Confluency was measured using time‐lapse microscopy using the Incucyte system (Mean ± *SD*, *n* = 3). Protein expression of MITF, phospho‐ERK, VINCULIN, Cleaved‐CASPASE3 and ERK in indicated cell lines under described treatment conditions (f). Growth curves of indicated cell lines with or without BRAFi (vemurafenib 0.5 µM) incubated in indicated CM from either hFOB 1.19 or osteoblasts, in the background of 40ng/ml RANKL nAb or siRNA mediated depletion of RANK (20nM). Confluency measured using time‐lapse microscopy and assessment of CASPASE3 activity assayed using cleavage activated Incucyte Dye reagent using the Incucyte system (Mean ± *SD*, *n* = 3) (g). Quantification of the population of A375 cells in S‐phase using EDU incorporation after 72 hr of treatment with 0.5 µM vemurafenib or DMSO in the presence or absence of 50ng/ml RANKL (Mean ± *SEM*, *n* = 3) (h). Quantification of the population of A375 cells in S‐phase using EDU incorporation after 72 hr of treatment with 0.5 µM vemurafenib or DMSO in the presence of CM from either hFOB 1.19 or osteoblasts. (Mean ± *SEM*, *n* = 6)

BRAFi‐treated melanoma cells co‐cultured with differentiated osteoblasts increased in cell number when compared to those co‐cultured with undifferentiated hFOB1.19 cells; an effect that was abolished by addition of a neutralizing antibody (nAb) to RANKL (Figure 5a, Figure S5a). A concomitant increase in MITF expression was observed in the osteoblast melanoma co‐cultures that was attenuated by addition of a RANKL nAb (Figure 5a, Figure S5a). An identical effect was observed when the RANK inhibitor SPD304, that blocks the trimerization of RANK and TNFα receptors (Douni et al., 2012), was substituted for the nAb (Figure 5b).

We next examined whether the increase in RANKL‐mediated survival occurred through inhibition of apoptosis. Indeed, co‐culture of A375 melanoma cells with osteoblasts inhibited the BRAFi‐induced cleavage of caspase 3 and counteracted the reduction in cell number, but the knockdown of RANK (RANK KD) was sufficient to re‐sensitize the co‐cultured melanoma cells to BRAFi (Figure 5c). Similar effects on caspase 3 activation were observed when RANKL signalling was inhibited with either SPD304 or RANKL nAb upon treatment of melanoma cells with osteoblast CM (Figure 5d) and conversely, treatment of melanoma cells with RANKL attenuated BRAFi induced caspase 3 activation (Figure S5b). Incubation of melanoma cell lines with osteoblast CM attenuated effects on cell numbers and caspase 3 activation induced by BRAFi in high (WM98, 501MEL) and low (A375) MITF expressing melanoma cell lines (Figure 5e‐f). This protective effect of osteoblast CM was decreased through the use of RANKL nAb, RANK inhibitor or RANK KD (Figure 5e‐f). The BRAFi prohibitive signalling from osteoblasts was however, unable to elicit protection in MITF negative melanoma cells (SKMEL105), indicating that intact MITF expression is required for osteoblast induced protection (Figure S5c). Finally, EdU incorporation assays demonstrate that the increase in melanoma cell number mediated by RANKL‐RANK signalling is not solely through pro‐survival effects, but rather contributes through an increase in proliferative capacity as shown by EdU incorporation (Figure 5g‐h). Taken together, these data indicate osteoblast‐derived RANKL alters the balance of death and proliferation that occurs when melanoma cells are treated with BRAF inhibitor to decrease apoptosis and increase cell‐cycle entry via upregulation of MITF.

## 4 DISCUSSION

Characterization of the complex mechanisms that govern metastatic growth in distant organs has been a goal for decades, ever since the “seed and soil” hypothesis was first postulated in 1889. In this study, we examined the role of the bone in fostering the growth and survival of melanoma. Bone stroma is composed of a network of cells working in concert to balance matrix deposition with destruction; chief among these cells are the osteoblasts. The relationship we have uncovered between osteoblasts and melanoma cells is one of mutuality; melanoma cells increase their proliferation when co‐cultured with osteoblasts, and osteoblasts show enhanced differentiation in the presence of a majority of melanoma cell lines. A similar relationship has been described for prostate and breast cancer cells that exhibit a predilection for forming bone metastases (Rahim et al., 2014).

Seifert et al have observed variable efficacy of melanoma responses to MAPK inhibitor therapy between metastatic sites, suggesting fundamental signalling differences between these sites (Seifert et al., 2016). Another possible explanation for this observation is that different sites for metastasis have different levels of drug bioavailability. However, preclinical in vivo studies of the BRAF‐V600E inhibitor vemurafenib find that drug accumulation occurs in both the liver and bones; niches that predict poor response in patients. As such, it is unlikely that bioavailability contributes to the poor response rates observed in melanoma bone metastases (Rissmann, Hessel, & Cohen, 2015). Thus, our finding that paracrine signalling via RANKL antagonizes BRAF inhibition is likely to have clinical significance.

RANKL does not drive tolerance to MAPK inhibition through pathway re‐activation as has commonly been observed (Lito et al., 2012; Smith et al., 2017; Straussman et al., 2012), but rather via an increase in MITF expression similar to the TNF‐α mediated increase in MITF that occurs when macrophages are co‐cultured with melanoma cells (Smith et al., 2014). It is now well established that elevated MITF expression mediates resistance to MAPK inhibitor based therapies (Haq, Shoag, et al., 2013; Haq, Yokoyama, et al., 2013; Johannessen et al., 2013; Rose et al., 2016; Smith et al., 2016, 2013, 2017). As such, RANKL signalling may be an “Achilles heel” of melanoma bone metastases that can be exploited therapeutically. A combinatorial approach using MAPK inhibition with the RANKL inhibitor Denosumab could, therefore, be employed specifically in patients displaying bone lesions.

The importance of RANKL/RANK signalling for bone niche maintenance, and previous reports demonstrating that melanoma migration and metastasis is driven by RANKL (Jones et al., 2006; Peinado et al., 2012), identifies RANK signalling as a candidate pathway worthy of further investigation in melanoma biology. We find that RANKL accelerates cell‐cycle progression, leading to enhanced proliferation and growth of melanoma cells. RANKL stimulation also leads to MITF up‐regulation with a subsequent enhancement of differentiation and survival likely due to activation of a MITF transcriptional program known to mediate these outcomes (Garraway et al., 2005; Johannessen et al., 2013; Miskolczi et al., 2018; Wellbrock & Arozarena, 2015). We found that in the TCGA melanoma cohort, elevated expression of the RANKL receptor RANK correlates with poor survival. This is an intriguing observation, though is likely due to a myriad of factors. In addition to the melanoma‐osteoblast interplay outlined in this study, RANKL signalling is important for lymphocyte differentiation and T‐cell activation; indeed inhibition of RANKL signalling has been shown to induce more anti‐tumour T‐cells in murine cancer models (Cheng & Fong, 2014; de Groot et al., 2018; Leibbrandt & Penninger, 2008). With immune checkpoint inhibitors becoming increasingly common in the clinic it is unsurprising that anti‐RANKL therapies have been proposed for melanoma, both alone and in combination with anti‐CTLA‐4 therapies (Ahern et al., 2017; Smyth, Yagita, & McArthur, 2016). Anti‐RANKL therapies may synergize not only with MAPKi, but also therapies that activate the adaptive immune system; the two standard of care approaches for treatment of disseminated melanoma.

How MAPK inhibition alters the bone microenvironment is poorly understood, and may contribute to the often‐poor drug responses observed in patients with bone metastasis. Indeed, stromal fibroblasts in BRAF inhibitor treated tumours alter the ECM and produce a more regressive micro‐environment for melanoma cells (Hirata et al., 2015). Further, MAPK inhibition may also alter osteoblast function. Although we have not addressed the role of MAPK signalling in osteoblasts specifically, we do find that co‐culture of melanoma cells with differentiated osteoblasts results in sufficient production of RANKL to antagonize MAPK inhibition. This observation implies that production of RANKL is not altered by the therapeutic intervention. Nevertheless, previous studies have implicated the MAPK pathway in osteoblast differentiation indicating that further studies maybe required to fully understand the impact of MAPK inhibition on the differentiation and production of RANKL from mature osteoblasts. Whilst we did not examine how melanoma cells stimulate osteoblasts to produce more RANKL, previous studies of other cancers such as prostate and breast have identified IL6 and PTHrP as RANKL stimulation factors. As both IL6 and PTHrP are expressed in some melanoma cell lines, this may be a conserved mechanism worthy of further investigation (Sottnik & Keller, 2013).

In conclusion, we have demonstrated that the RANKL‐RANK signalling that occurs between osteoblasts and melanoma cells drives the proliferation, differentiation and survival of melanoma cells. Osteoblast‐derived RANKL stimulates MITF‐driven tolerance to MAPK inhibition, which may contribute to the increased resistance to targeted therapies observed in melanoma patients with metastatic bone lesions.

## CONFLICT OF INTEREST

The authors declare that they have no conflict of interest.
