# Supplementary material for: Osteoblasts contribute to a protective niche that supports melanoma cell proliferation and survival
Source: Pigment Cell Melanoma Res. 2019 Aug 8;33(1):74–85. doi: 10.1111/pcmr.12812 (PMC6972519; doi:10.1111/pcmr.12812)
Supplement: Supplementary file 1 [file PCMR-33-74-s001.pdf]

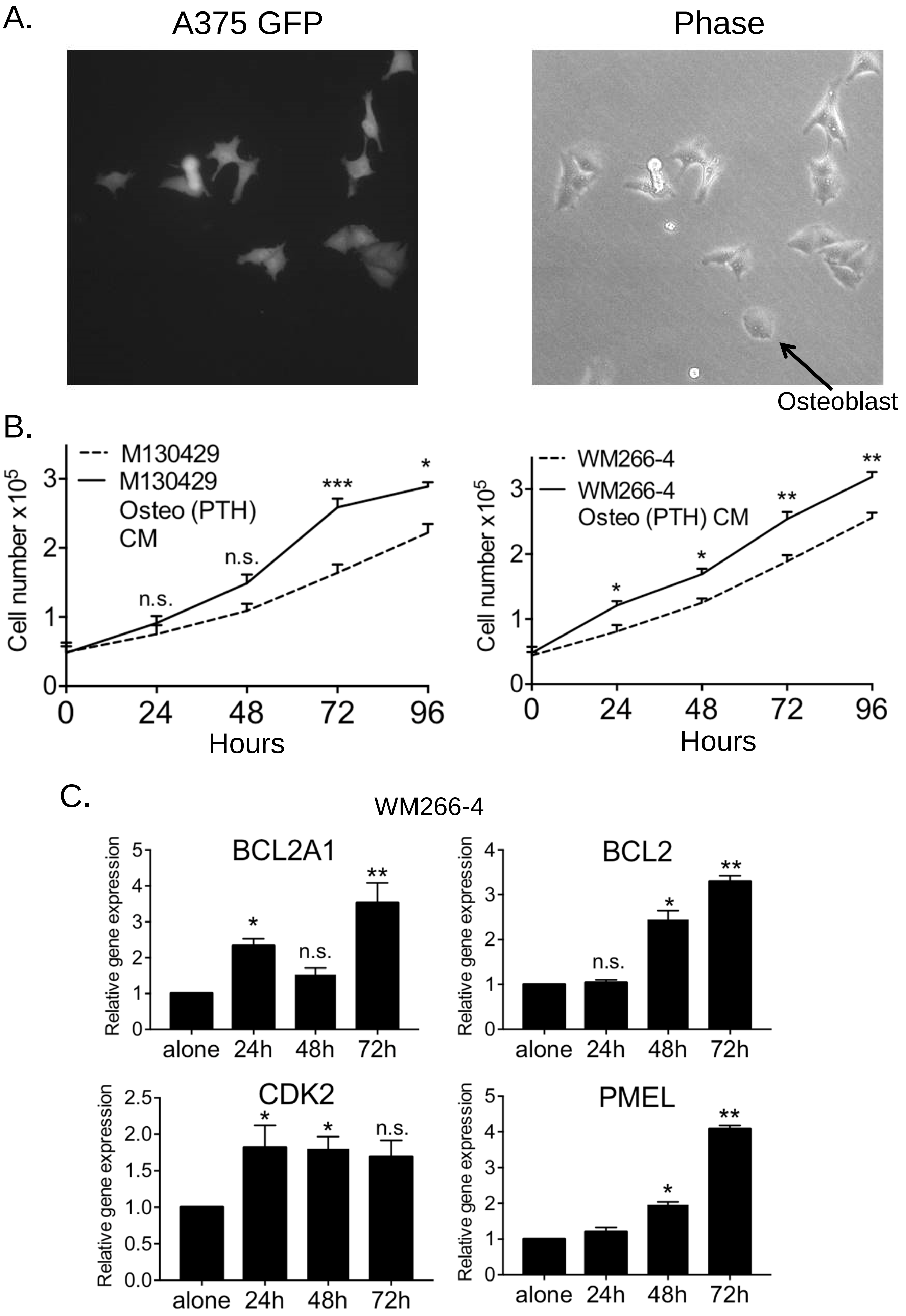

Supplemental Figure 1: Additional osteoblast induced changes to melanoma

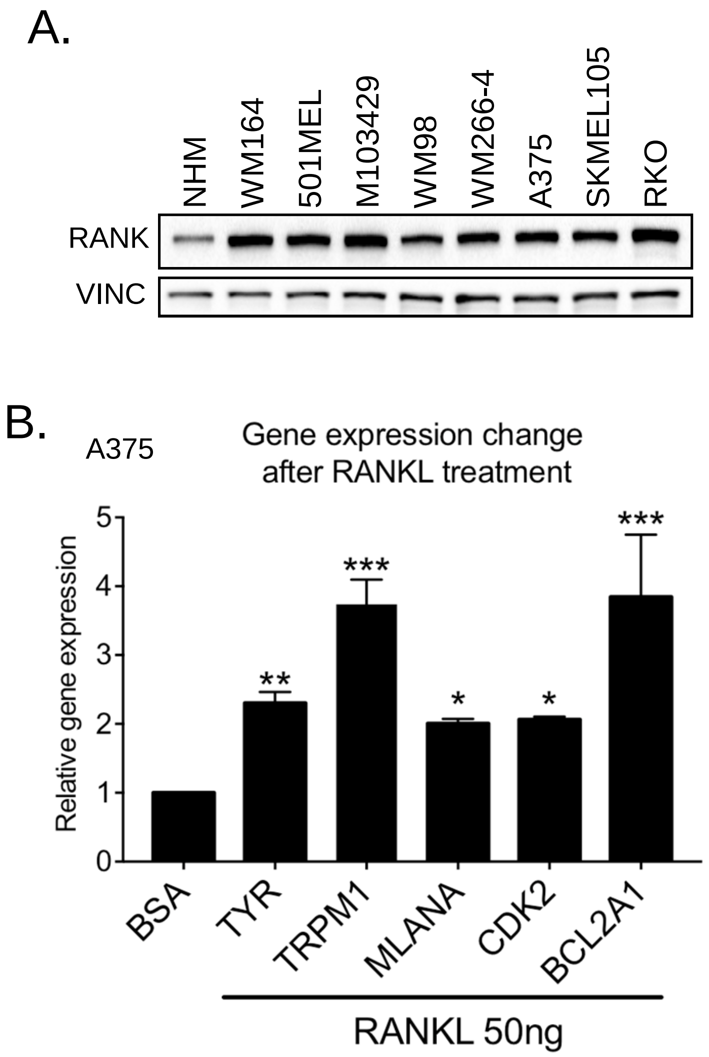

Supplemental Figure 2: Additional effects of RANKL on MITF target genes

A.

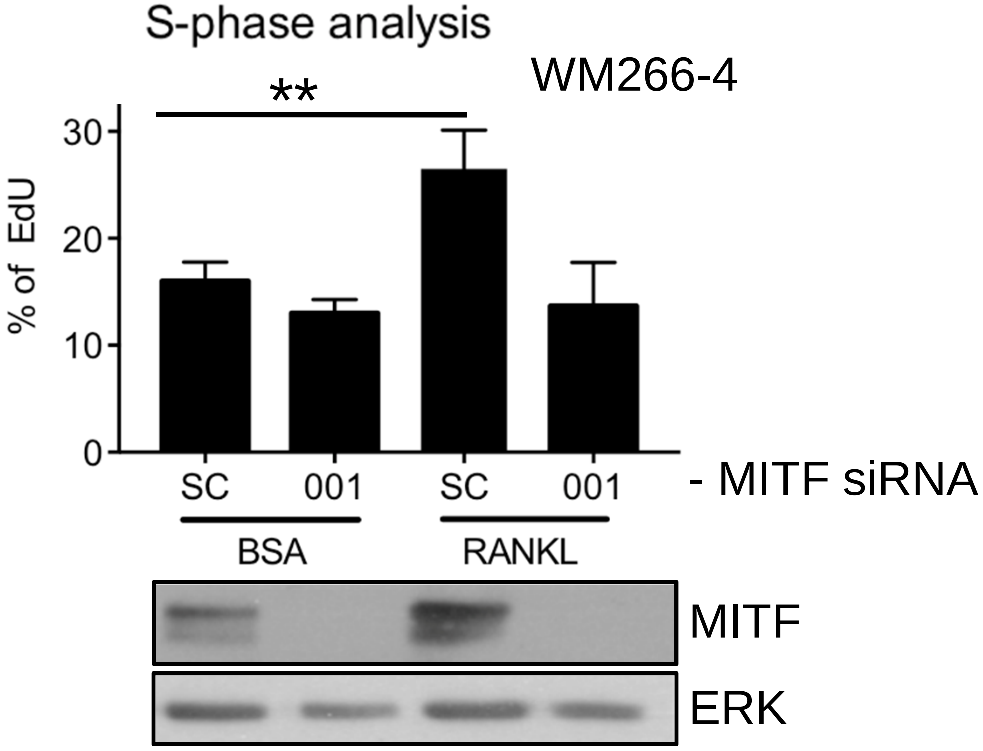

Supplemental Figure 3: MITF drives RANKL induced proliferation

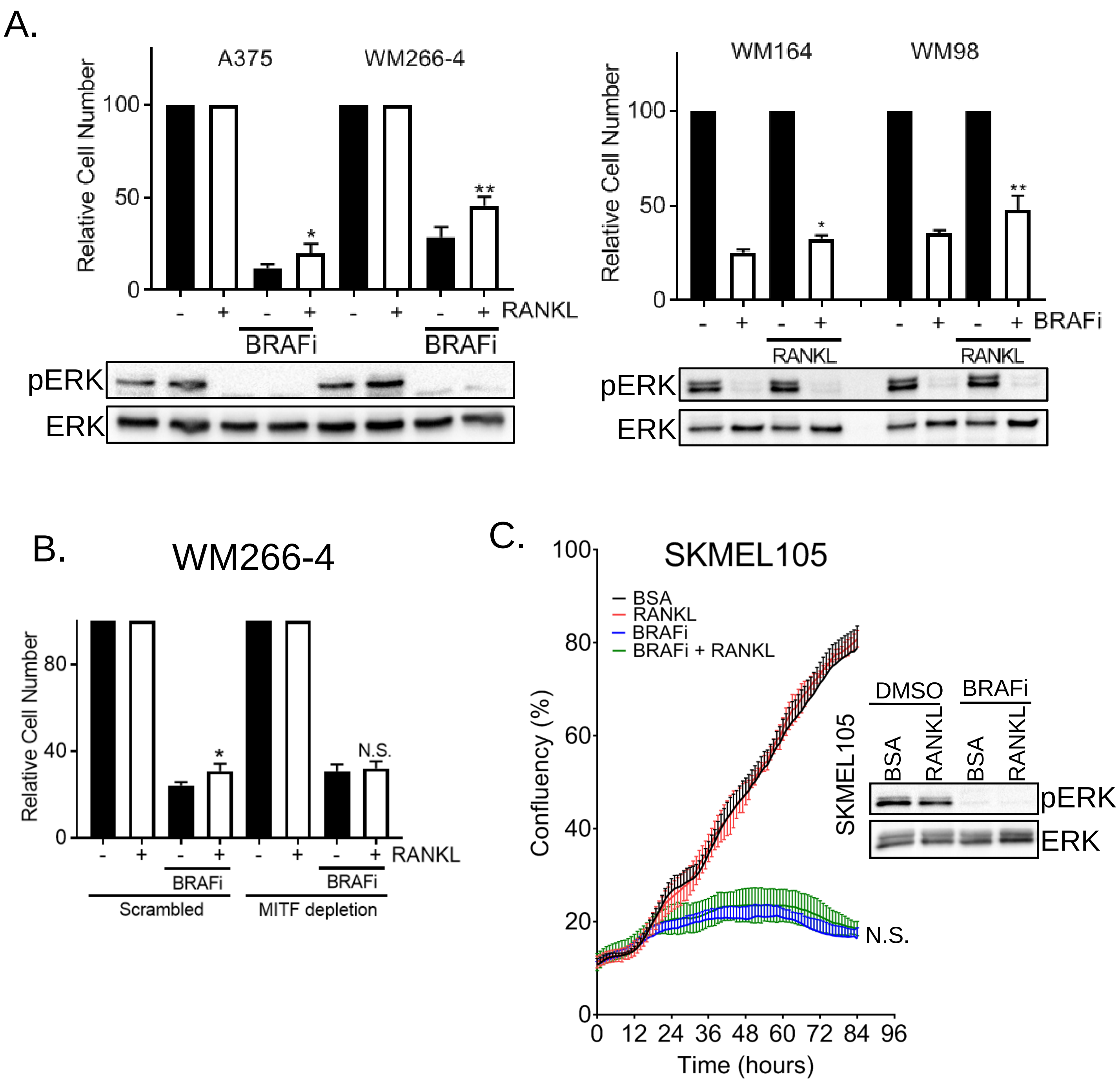

Supplemental Figure 4: Additional changes of sensitivity to BRAF inhibition in BRAF mutant cells

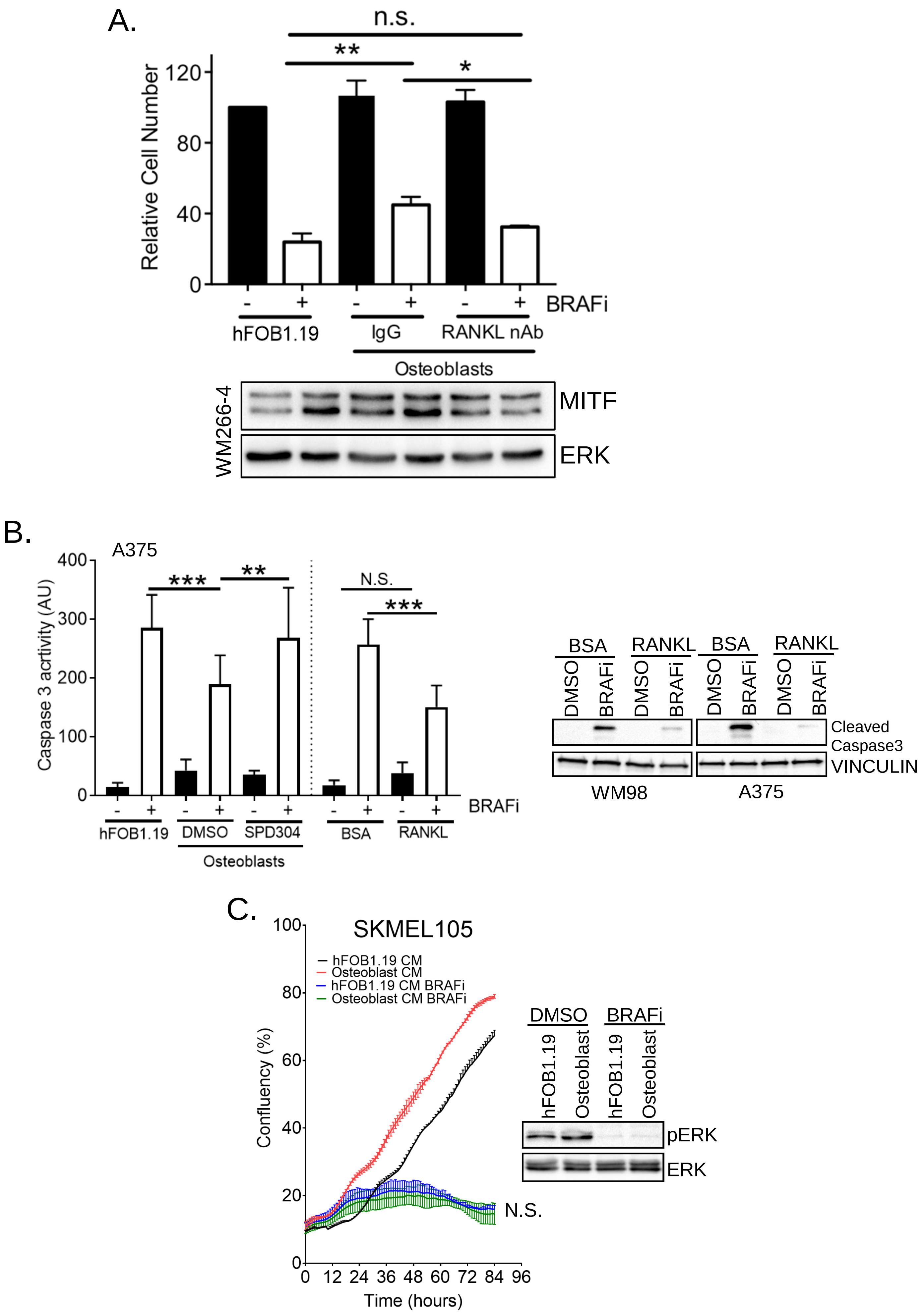

Supplemental Figure 5: Additional osteoblast-melanoma co-culture survival data
